# Life Science 2.0: reframing the life science sector for ‘the benefit on mankind’

**DOI:** 10.1080/16549716.2024.2330758

**Published:** 2024-04-05

**Authors:** Michaela Vallin, Göran Tomson, Beate Kampmann, Eivind Engebretsen, Stefan Swartling Peterson, Rhoda K. Wanyenze, Ole Petter Ottersen

**Affiliations:** a Chemical Biology Consortium Sweden, SciLifeLab, Department of Medical Biochemistry and Biophysics, Karolinska Institutet, Stockholm, Sweden; b Department of Learning, Informatics, Management, and Ethics, Karolinska Institutet, Stockholm, Sweden; c Charité Centre of Global Health, Charité Universitätsmedizin, Berlin, Germany; d Faculty of Medicine, University of Oslo, Oslo, Norway; e Department of Global Public Health, Karolinska Institutet, Stockholm, Sweden; f College of Health Sciences, School of Public Health, Makerere University, Kampala, Uganda; g Sustainable Health Unit and Institute of Basic Medical Sciences, University of Oslo, Oslo, Norway; h Clinical Neuroscience, Karolinska Institutet, Stockholm, Sweden

**Keywords:** Biomolecular R&D, life science sector, health for all, COVID-19, Nobel Prize 2023, societal value, knowledge sharing, health equity, global governance, sustainable health

## Abstract

The COVID-19 pandemic put the life science sector to the test. Vaccines were developed at unprecedented speed, benefiting from decades of fundamental research and now honoured by a Nobel Prize. However, we saw that the fruits of science were inequitably distributed. Most low- and middle-income countries were left behind, deepening the inequalities that the Sustainable Development Goals were set to reduce. We argue that the life science sector must reinvent itself to be better and more equitably prepared for the next health crisis and to ensure fair access to health across current and future generations. Our recommendations include global governance, national strategies and the role of universities and corporations. Improved and more equitable health care should be centre stage for global health action and a core mission of a reframed Life Science sector – what we call Life Science 2.0.

**Paper Context**
- **Main findings**: During the COVID-19 pandemic the Life Science sector stepped up to the challenge, but vaccines and medicines were not equitably distributed.
- **Added knowledge**: Obstacles were identified that hindered global access to medical innovations.
- **Global health impact for policy and action**: Global and national governance, universities and the private sector should join forces to create a Life Science sector (Life Science 2.0) that affords equitable access to medical advances across geographical and generational boundaries and socio-economic strata.

The COVID-19 pandemic unveiled strengths and weaknesses of the life science sector – here broadly defined as the entire set of industrial, academic, and healthcare actors and relevant expert authorities and governance bodies that engaged in the response to one of the most serious health crises that the world has ever seen. We observed how the pandemic put the life science sector to the test and radically intensified collaboration between the different actors and between academia and industry in particular. This led to the development of new vaccines and treatments at an unprecedented speed – a progress that benefited from and depended upon decades of publicly funded research and that capitalised on targeted investments. In the midst of a health crisis, we have witnessed a medical success story.

However, the rewards of this success story were unevenly and unjustly distributed [1]. It became blatantly clear that large parts of the world are precluded from enjoying the fruits of science even during a health crisis. Most low- and middle-income countries (LMICs) were left behind while vaccines and medicines were retained and even hoarded elsewhere. Clearly, knowledge and products based on public spending were not made available to society at large [2,3].

In light of these disparities in global health, the 2023 Nobel Prize in Physiology or Medicine takes on special significance. The Prize was awarded to Katalin Karikó and Drew Weissman ‘for the discoveries concerning nucleoside base modifications that enabled the development of effective mRNA vaccines against COVID-19.’ Their work not only represents an exemplary case of how basic research can lead to significant societal benefits but also highlights the importance of equitable access to life-saving technologies and health care. Furthermore, the Prize brings to the fore the breakthroughs in basic research that paved the way for the rapid development of mRNA vaccines.

The development of these vaccines was firmly anchored in knowledge generated by state-funded basic research on the genetic code, viruses, cell biology, immunology, PCR technology, genome sequencing, and research on lipids. We only need a quick look at the list of Nobel Prizes to realise why we were better equipped to handle COVID-19 than the Spanish flu. It was good fortune that the mRNA technology was ready for its debut when COVID-19 hit.

According to Alfred Nobel’s will, the Prize should be awarded ‘ … to those who, during the preceding year, shall have conferred the greatest benefit on mankind.’ The discoveries made by Karikó and Weissman were crucial for the vaccine development we have witnessed. Yet, while the mRNA vaccines have saved millions of lives, they were inequitably distributed and thus did not serve their full potential in benefiting *mankind* [1,3]. The practice among rich countries to purchase and hoard more vaccines than they needed limited and delayed the availability of vaccines for other countries [3,4]. In addition, within countries with access to vaccines, there were disparities in vaccination rates based on socioeconomic factors, leading to suboptimal societal value [5–10]. Thus, there is a clear need to reframe the life science sector. We have an ethical obligation to ensure that essential medicines, including life-saving drugs and vaccines, are affordable and accessible to all, regardless of economy or geography [11]. To achieve a just distribution of vaccines and medicines in and between crises, radical changes are needed. We must build capacity for vaccine and medicine production and uptake also in LMICs. Efforts to strengthen the life science value chain are required – from research and development, through production, quality control, and clinical trials, to distribution and market pull. Education and training across the spectrum of product development and implementation are essential [12,13].

In the wake of the COVID-19 pandemic and inspired by the recent Nobel Prize in Physiology or Medicine, we should uphold the collaborative spirit of the life science sector but also mend its weaknesses. As a key element in global health action, all stakeholders should help mold a new life science mission for the post-pandemic era [14] – what we call Life Science 2.0.

Universal preparedness for health (UPH) [15,16] is at the core of Life Science 2.0 and refers to a comprehensive approach to protect – in an equitable manner – communities and individuals from infectious disease outbreaks, natural disasters, or other public health threats. It involves strengthening health systems, building capacity for early detection and response to emerging threats, promoting access to essential medicines and vaccines, and improving communication and coordination among different stakeholders involved in emergency preparedness and response. UPH also emphasises the importance of addressing the underlying social, economic, and environmental factors [17,18] that contribute to health risks, such as poverty, inequality, climate change, and urbanisation.

The move towards Life Science 2.0 should be driven by the conviction that the knowledge we generate at our universities – the fruits of science – should come to the benefit of all, by securing access to medicines and vaccines also in low-resource settings.

## Building Life Science 2.0

We provide the following five recommendations for building Life Science 2.0:

1. **Global governance must prioritise sustainable tech transfer, capacity building, and public trust in under-resourced regions**. *The global governance system* must see it as an issue of paramount importance to create the market mechanisms and market pull that are required to sustain and improve current tech transfer initiatives in resource-poor settings. The new leadership in the World Bank should consider this a top priority. The Coalition for Epidemic Preparedness Innovations (CEPI) sets an example targeted at vaccines [19]. Solutions must be identified to avoid health being impacted by inflexible intellectual property (IP) policies. Initiatives exist to license and innovate more efficiently [20,21], but the complexity associated with scaling innovations remains. WTO’s partial patent waiver for COVID-19 vaccines [22] recognises that IP flexibility facilitates increased local capacity and globally decentralised supply chains of immediate and future benefit. In addition, open procurement and disclosure build necessary public trust in the decision-making process.
2. **National life science strategies must focus on preparedness, equity, and sustainability**. When generating strategies for life science, *national governments* in high-income countries must see the life science sector as a lever for preparedness, equity, and sustainability and not only as a platform for economic prosperity and better health for those who live today. In LMICs, strengthened government capacity in working with the private sector is needed to achieve local implementation of global health goals [23].
3. **Universities, as hubs of innovation and education, are key to fostering health preparedness in low-resource settings**. As arenas for critical reflection, innovation, education, multidisciplinarity, and system thinking, *universities* are uniquely positioned to develop the holistic approaches that are needed to build preparedness for health in low-resource settings [3]. Universities may provide a platform for a whole-of-society approach [24] and serve as breeding grounds for new ideas on removing obstacles that have hindered so many from benefitting from the progress of science [25]. There is both a political and public need for scientists to do better as knowledge brokers. To rise to the challenge of the increased misinformation and distrust that significantly impede uptake of medicines and vaccines should be considered as an integral part of the universities´ third mission. Academia should provide evidence, teach critical reflection and scientific literacy, and promote open science to benefit future preparedness.
4. **Addressing the global health inequity: a call to action for corporations**. Investors, customers, employees, and future talents expect *corporate actors* to address the extant inequities that preclude prompt distribution of products to resource poor settings. A case in point is Africa, currently importing 95% of its medicines and 99% of the vaccines needed on the continent [26,27]. Current tech transfer initiatives should be emulated and intensified, in close cooperation with WHO [28] and the academic sector. The WHO and the Medicines Patent Pool established in 2021 a Technology Transfer Programme for mRNA vaccines in South Africa [20] and Rwanda will be the first African country to host an autonomous mRNA manufacturing facility [27]. This bodes well for future efforts towards UPH. The private sector must engage with global and national governance systems to identify a business model that makes societal value a common goal. For example, the Advance Market Commitment (AMC) model [29] created by Nobel Laureate Michael Kremer points to a way forwards. This initiative is a funding mechanism to incentivise private actors to develop products for low-resource markets. An AMC model that moves investor commitment downstream by outcome-based payments can incentivise research priorities and capacity building that creates direct societal value and be applied to long-term health challenges.
5. **Embracing knowledge as a global public good is a shared responsibility among life science stakeholders**. *All life science stakeholders* must see knowledge as a global public good and recognise a shared responsibility for fair implementation and long-term needs. Coordinated policy development approaches across disciplines and integration at national – regional – global levels are needed, as is the system-wide accountability [11,28].

## Taking joint responsibility for Life Science 2.0

The multidisciplinary and multisectoral nature of life science brings a shared responsibility for translating knowledge into better health for all [20,25]. Traditionally, different actors consider themselves responsible for different parts of the value chain: academics commonly view education and basic research as their main responsibilities. Academic entrepreneurs and small enterprises alike, often strive for refining a project to the point where big pharma finds interest to incorporate it into their project portfolio. The industry focuses on research and bringing the product to market. However, the universities´ ‘third mission’ says that the responsibility of universities extends beyond teaching and research [30] while industrial actors are expected to embed environmental, social, and governance perspectives (ESG) [31] in their strategies and operations. In Life Science 2.0 the ESG principles should be redefined with a focus on UPH as a crucial component of sustainability. While ESG pays increasing attention to human rights in supply chains, it seems paradoxical not to include human rights obligations also in regard to access to the final products.

Reframing life science – Life Science 2.0 – means that all actors should take a long-term and global public health perspective, ensuring preparedness across generational and geographical boundaries and across socioeconomic strata. This would be perfectly aligned with the ambitions embedded in UN´s Sustainable Development Goals and with the lessons learned from the COVID-19 pandemic. The key is to preserve the collaborative spirit that fostered such rapid medical progress during the pandemic, but at the same time recognise the need to secure a more equitable and robust preparedness. More than ever should life science actors see themselves as part of a sector with a joint responsibility for the health of future generations. Indeed, we should be faithful to the definition of life science as ‘an interdisciplinary research branch that deals with the study of biological life as well as internal and external conditions for continued life’ [32]. To secure ‘continued life’, human health must be seen in the context of animal and planetary health and with a time horizon that recognise our responsibility for future generations.

In summary, the COVID-19 pandemic has unveiled the strengths of the life science sector but has also revealed and deepened health inequities that plainly demonstrate how far we are from a fair distribution of the achievements of research and innovation. Inspired and compelled by the fresh lessons of the pandemic and the most recent Nobel Prize in Physiology or Medicine, now is the time to mobilise and reframe the life science sector so that we can serve humankind at large, across geographical, socioeconomic, and generational boundaries. Actors must join forces to address all steps from research to societal value and help ensure that preparedness be based on solid footing in those communities and countries that were currently left behind. This should be a call to global health action and a major mission of the post-pandemic life science sector – Life Science 2.0.
